# Positive effects of bubbles as a feeding predictor on behaviour of farmed rainbow trout

**DOI:** 10.1038/s41598-022-15302-7

**Published:** 2022-07-05

**Authors:** Aude Kleiber, Jean-Michel Le-Calvez, Thierry Kerneis, Axel Batard, Lionel Goardon, Laurent Labbé, Valentin Brunet, Vitor Hugo Bessa Ferreira, Vanessa Guesdon, Ludovic Calandreau, Violaine Colson

**Affiliations:** 1 JUNIA, Comportement Animal et Systèmes d’Elevage, 59000 Lille, France; 2 INRAE, PEIMA, 29450 Sizun, France; 3 IFM Biology, AVIAN Behavioural Genomics and Physiology Group, Linköping University, 58183 Linköping, Sweden; 4 INRAE, CNRS, IFCE, Université de Tours, Centre Val de Loire UMR Physiologie de La Reproduction et des Comportements, 37380 Nouzilly, France; 5 INRAE, LPGP, Campus de Beaulieu, 35042 Rennes, France

**Keywords:** Feeding behaviour, Learning and memory, Classical conditioning, Emotion, Aggression, Animal behaviour, Attention, Perception

## Abstract

Occupational enrichment emerges as a promising strategy for improving the welfare of farmed animals. This form of enrichment aims to stimulate cognitive abilities of animals by providing them with more opportunities to interact with and control their environment. Predictability of salient daily events, and in particular predictability of feeding, is currently one of the most studied occupational enrichment strategies and can take several forms. In fish, while temporal predictability of feeding has been widely investigated, signalled predictability (based on a signal, such as light or sound) has received little attention. Depending on the type of predictability used and the ecology of the species, the effects on fish welfare often differ. The present study aimed to determine which feeding predictability would be most appropriate for rainbow trout, the main continental farmed fish in Europe, and what the consequences might be for their welfare. We tested four feeding predictability conditions: temporal (based on time of day), signalled (based on bubble diffusion), temporal + signalled (based on time and bubble diffusion), and unpredictable (random feeding times). Behavioural and zootechnical outcomes recorded were swimming activity, aggressive behaviours, burst of accelerations, and jumps, emotional reactivity, and growth. Our results showed that rainbow trout can predict daily feedings relying on time and/or bubbles as predictors as early as two weeks of conditioning, as evidenced by their increased swimming activity before feeding or during feed omission tests, which allowed to reinforce their conditioned response. Temporal predictability alone resulted in an increase in pre-feeding aggressive behaviours, burst of accelerations, and jumps, suggesting that the use of time as the sole predictor of feedings in husbandry practices may be detrimental to fish welfare. Signalled predictability with bubbles alone resulted in fewer pre-feeding agonistic behaviours, burst of accelerations, and jumps than in the temporal predictability condition. The combination of temporal and signalled predictability elicited the highest conditioned response and the level of pre-feeding aggression behaviours, burst of accelerations and jumps tended to be lower than for temporal predictability alone. Interestingly, fish swimming activity during bubble diffusion also revealed that bubbles were highly attractive regardless of the condition. Rainbow trout growth and emotional reactivity were not affected by the predictability condition. We conclude, therefore, that the use of bubbles as a feeding predictor could represent an interesting approach to improve rainbow trout welfare in farms, by acting as both an occupational and physical enrichment.

## Introduction

A potential way to improve welfare of captive animals is to give them opportunities to actively interact with their environment by meeting moderate challenges, controlling some aspects of the environment, or anticipating specific events, thereby mobilizing their cognitive skills. This practice aims to improve (or enrich) housing conditions and is called occupational (or cognitive) enrichment^1,2^. Allowing captive animals to predict positive (e.g., feeding) and/or aversive events (e.g. handling) by the use of classical conditioning paradigms offers them the opportunity to gain greater knowledge about their environment, to be more prepared, and to better cope with possible environmental changes^3–5^. Different types of predictability (i.e., having information about the regularity of salient daily events^6^) exist based on two main types: (i) temporal predictability, when an event occurs at fixed time-intervals (temporally predictable); (ii) signalled predictability, when a signal (CS, conditioned stimulus) precedes a positive or a negative event (US, unconditioned stimulus) eliciting a spontaneous response. Temporal predictability relies on the animal’s ability to synchronize their behavioural and physiological processes for the different events they encounter in their environment^7^. This kind of predictability is inherent to the biological clock of the animal and is thus potentially longer to appraise than signalled predictability which is based on the animal’s success in classical conditioning.

Most studies of the effects of predictability for captive animals have focused on one particular positive event, feed delivery. These studies showed that this predictability elicited a feed anticipatory activity (FAA), a possible indicator of positive emotions due to the activation of the reward neural circuits^6,8^.

In fish, few studies have so far investigated the welfare effects of feed anticipation, with temporal predictability being the most studied condition. For example, better growth was observed for different fish species (seabream, *Sparus aurata*^9^, seabass, *Dicentrarchus labrax*^10^, and Atlantic cod, *Gadus morhua*^11^), when feedings were distributed at a fixed time schedule for 2 to 4.5 months. For the Atlantic cod, it was also found that temporal predictability reduced fish fin damages^11^. Furthermore, temporal predictability induced FAA prior to feed delivery, associated with reduced total swimming activity during unfed periods for the seabream^9^. However, temporal predictability has also been reported to induce aggressive behaviors in salmons (*Salmo salar*) and no effect on body growth^12^.

In contrast to temporal feeding predictability, signalled feeding predictability in fish husbandry, and its effects on animal welfare, has been poorly studied. Many CS have already been shown to successfully condition fish to feeding, such as a light^13–17^, a card^18^, the water level^19^, or a sound^20,21^, but their effects on fish welfare were minor or not investigated. By using classical conditioning for two weeks, researchers showed that signaling feed delivery by a CS (light) decreased cortisol levels and led to a positive emotional-like state in seabream^17^. Conversely, giving tilapia (*Oreochromis mossambicus*) the possibility to anticipate feedings by a visual cue (a yellow and black stripped card) for three weeks seemed to induce higher plasma cortisol after an acute stress^18^. In salmons, two-month of light conditioning for feeding led to no or negative effects on growth parameters^16^. These discrepancies between studies may depend on the ecology of the species and the type or relevance of stimulus used. Furthermore, the varying effects observed between temporal and signalled predictabilities may be related to the contradictory roles played by the inherent biological clock of the fish and a neutral (and possibly irrelevant) stimulus warning the imminence of the feed delivery. Unlike captive mammals, few studies have been conducted in fish to conclude a clear effect of feeding predictability on welfare^22^. Further research is therefore needed to identify the most appropriate occupational enrichments for farmed fish.

To broaden our knowledge on which type of predictor (temporal and/or signalled) is the easiest for farmed fish to learn and the welfare consequences of these occupational enrichments, we studied the rainbow trout (*Oncorhynchus mykiss*), the main farmed continental fish in Europe^23^ as the animal model. Four feeding predictability conditions were applied over two weeks: temporal (based on time of day), signalled (based on bubble diffusion), temporal + signalled (based on time and bubble diffusion), and unpredictable (random feeding delivery). Bubble diffusion was chosen as a conditioned stimulus (CS) for its interesting properties, because in addition to being visible and audible to all fish in the tank at the same time, it also provides tactile stimulation to the fish and could easily be applied in fish farms. For each condition, fish group swimming activity was recorded prior to feed delivery to assess the efficiency of each predictor. Then, several welfare indicators were observed during the experimental period: aggressive behaviours, burst of accelerations and jumps, emotional reactivity, stress-induced anorexia—all tending to indicate poor welfare when increased -, and better growth indicating good welfare. At the end of the two-week period, feed omission tests were performed to evaluate fish activity during omissions and frustration, as measured by aggressive behaviours^24,25^.

## Results

### Weight and size of fish

Fish weight (Mean ± SEM: BUBBLE + TIME: 9.03 ± 0.37 g; BUBBLE: 9.89 ± 0.36 g; TIME: 9.56 ± 0.31 g; RANDOM: 9.75 ± 0.45 g), body size (Mean ± SEM: 8.79 ± 0.16 cm; 9.21 ± 0.12 cm; 9.12 ± 0.14 cm; 9.11 ± 0.17 cm, respectively) and K-factor (Mean ± SEM: 1.33 ± 0.04; 1.26 ± 0.02; 1.27 ± 0.03; 1.28 ± 0.03, respectively) were not significantly different between treatments at the end of the experiment (*P* = 0.40; *P* = 0.19; *P* = 0.36 respectively).

### Group behaviours during the 12 days of conditioning

**Figure 1:**
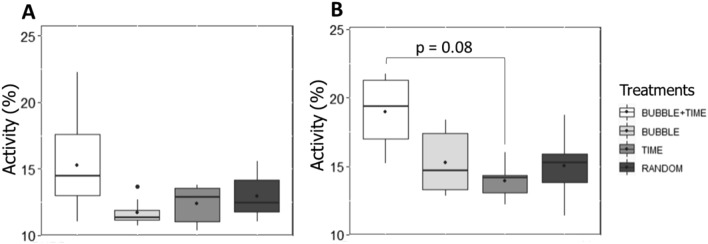
Median (quartiles: 25%, 75%) percentage of fish group activity (% of differing pixels between two successive images) on (**A**) day 1 and on (**B**) day 12 of conditioning. Activity is measured in the bubble area during the 15 s of bubbles until the 5 s of time gap after bubbles for all treatments, corresponding to the period before the expected feeding for treatments BUBBLE + TIME and BUBBLE (total analysis duration of 20 s). The black point inside boxes represents the mean value (Tukey post-hoc tests).

On day 1, trout exhibited similar activity in response to bubble diffusion into the bubble area, regardless of their treatment (LMM: *P* = 0.13; Fig. 1A). Independently of treatments, group swimming activity (see methods section for variable description) was higher during the last bubble diffusion compared to the first one on day 1 (LMM, χ^2^ = 10.71, df = 2, *P* = 0.005; Tukey, *P* = 0.01).

On the last day of conditioning (day 12), we found a significant effect of the treatment (LMM, χ^2^ = 9.31, df = 3, *P* = 0.03; Fig. 1B) and the moment when bubbles were diffused (LMM, χ^2^ = 10.29, df = 2, *P* = 0.006) on group activity. However, post-hoc analyses did not reveal any significant difference between treatments (Tukey, all *P* > 0.05), except a tendency for higher activity in BUBBLE + TIME fish than in TIME fish (Tukey, *P* = 0.08; Fig. 1B).

**Figure 2:**
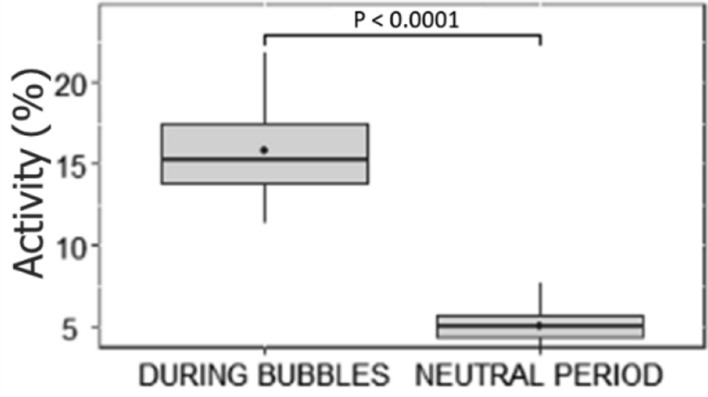
Median (quartiles: 25%, 75%) percentage of fish group activity (% of differing pixels between two successive images) in the bubble area on the day 12 of conditioning. “During bubbles” represents the 15 s of bubble diffusion + 5 s of time gap for all treatments. “Neutral period” represents 6-min periods with no feed delivery or bubble diffusion. The black point inside boxes represents the mean value (Tukey post-hoc test).

On day 12, the last bubble diffusion induced an increased activity compared to the first ones (Tukey, first-last: *P* = 0.05; third-last: *P* = 0.02). The absence of a significant higher activity in BUBBLE and BUBBLE + TIME compared to TIME and RANDOM on the last day of conditioning was probably due to a strong attractiveness of the fish for bubbles in all treatments. Indeed, group activity was significantly higher during bubble diffusion than during neutral periods (i.e., 6-min period with no feed delivery or bubble diffusion) into the bubble area, independently of treatments (LMM, χ^2^ = 1846.10, df = 1, *P* < 0.001; Fig. 2).

**Figure 3:**
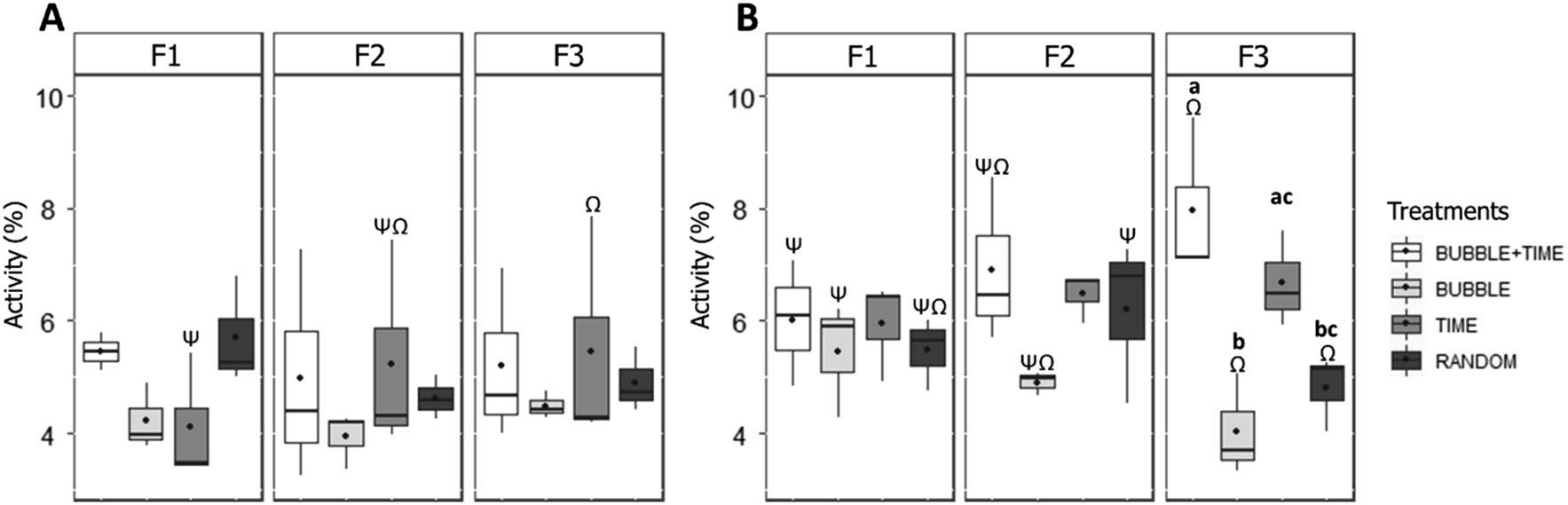
Median (quartiles: 25%, 75%) percentage of fish group activity (% of differing pixels between two successive images) in the whole tank during the 6-min period preceding feed delivery. Results are given for each treatment and for the three feedings on (**A**) day 1 and (**B**) day 12 of conditioning. F1, F2 and F3 correspond to the three feedings analysed. The black point inside boxes represents the mean value. Different latin letters indicate significant differences between treatments, and different greek letters indicate differences between feedings (*P* < 0.05; Tukey post-hoc tests).

On day 1, analysis of group activity in the whole tank for 6 min preceding feedings showed no significant difference between treatments (Fig. 3A). TIME fish exhibited a significantly higher activity prior to the last feeding (feeding 3) compared to feeding 1 (interaction treatment x feeding number: LMM: χ^2^ = 20.35, df = 11, *P* = 0.04; Tukey, *P* = 0.03; Fig. 3A).

On day 12, group activity during this 6-min period was both dependent on the treatment and on the feeding (interaction group x feeding number: LMM: χ^2^ = 28.90, df = 6, *P* < 0.001; Fig. 3B). On this day, the two conditions where fish could learn to anticipate feeding using time as a predictor (BUBBLE + TIME and TIME) exhibited a higher activity prior to feeding 3 than in BUBBLE fish (Tukey, *P* = 0.002; *P* = 0.03 respectively; Fig. 3B). BUBBLE + TIME fish also exhibited a higher activity prior to feeding 3 than RANDOM fish (Tukey, *P* = 0.009), indicating that both treatments (BUBBLE + TIME and TIME), at the end of the conditioning, did anticipate feed delivery using time as predictor, with BUBBLE + TIME fish being the most temporally conditioned (Fig. 3B).

Analysis of the frequency of agonistic behaviours, burst of accelerations, and jumps during the 6 min preceding feeding revealed an independent effect of the day (GLMM: χ^2^ = 10.89, df = 1, *P* < 0.001) and the treatment (GLMM: χ^2^ = 17.22, df = 3, *P* < 0.001). The effect of days revealed that fish elicited significantly higher agonistic interactions, burst of accelerations, and jumps at day 1 compared to day 12 of conditioning (Tukey, *P* < 0.001).

**Figure 4:**
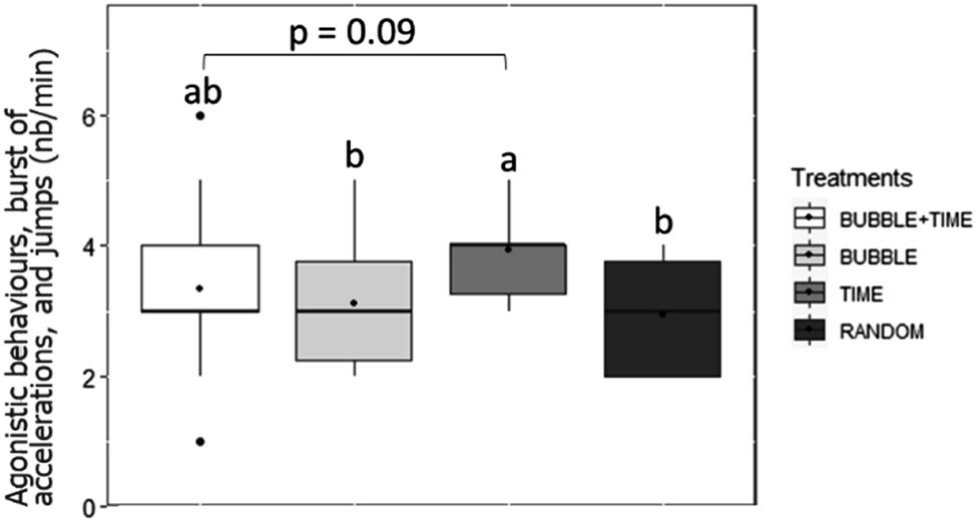
Median (quartiles: 25%, 75%) number of agonistic behaviours, burst of accelerations, and jumps scored per minute in the whole tank over 6 min preceding feed delivery in the four treatments. The black point inside boxes represents the mean value. Different letters indicate significant differences between treatments (*P* < 0.05; Tukey post-hoc tests).

The effect of the treatment revealed that TIME fish expressed significantly higher agonistic behaviours, burst of accelerations, and jumps than fish from BUBBLE (Tukey, *P* = 0.02) and RANDOM (Tukey, *P* < 0.001). Although non-significant, the same tendency was observed between treatments TIME and BUBBLE + TIME (Tukey, *P* = 0.09; Fig. 4).

### Feed omission test

**Figure 5:**
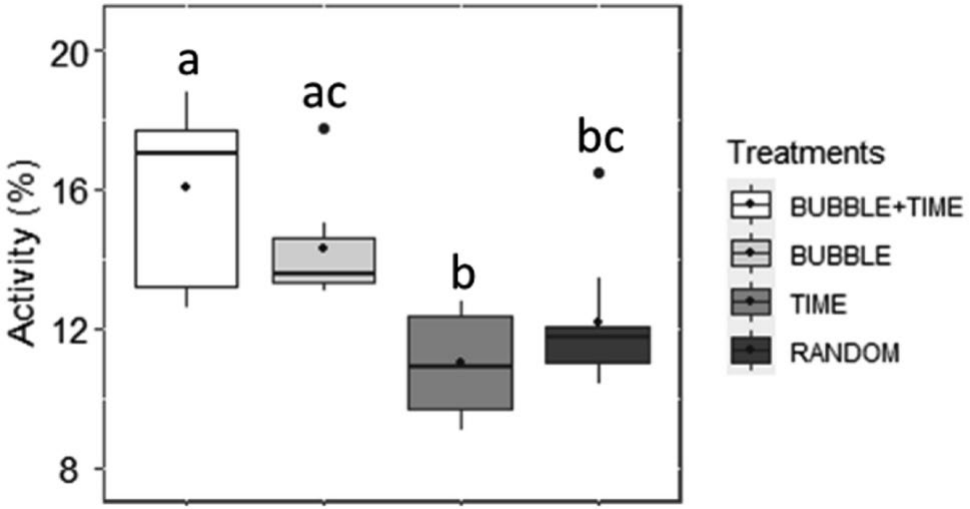
Median (quartiles: 25%, 75%) percentage of fish group activity (% of differing pixels between two successive images) during feed omissions. Activity is measured in the bubble area during bubble diffusion to 30 s after bubbles (total analysis duration of 45 s: 15 s of bubbles, the 5 s of time gap before the expected feeding for treatments BUBBLE + TIME and BUBBLE, and 25 s after the omission. For treatments TIME and RANDOM, the period of 25 s started 5 s after the end of bubbles). The black point inside boxes represents the mean value. Different letters indicate significant differences between treatments (*P* < 0.05; Tukey post-hoc tests).

Although analyses conducted during conditioning did not allow to show that trout learned to use bubbles as a predictor of feeding, feed omissions did. Analysis of group activity on the three omission occasions, during the period that included the 15 s of bubble diffusion, the 5 s of time gap before the expected feeding for treatments BUBBLE + TIME and BUBBLE, and the 25 s that started 5 s after the end of bubbles for all treatments, revealed a treatment effect only (LMM: χ^2^ = 30.33, df = 3, *P* < 0.001). During feed omissions, activity measured in the bubble area was higher in BUBBLE + TIME and in BUBBLE than in TIME (Tukey, *P* = 0.005 and *P* = 0.02 respectively, Fig. 5). Furthermore, the activity measured in BUBBLE + TIME was higher than in RANDOM (Tukey, *P* = 0.03; Fig. 5).

**Figure 6:**
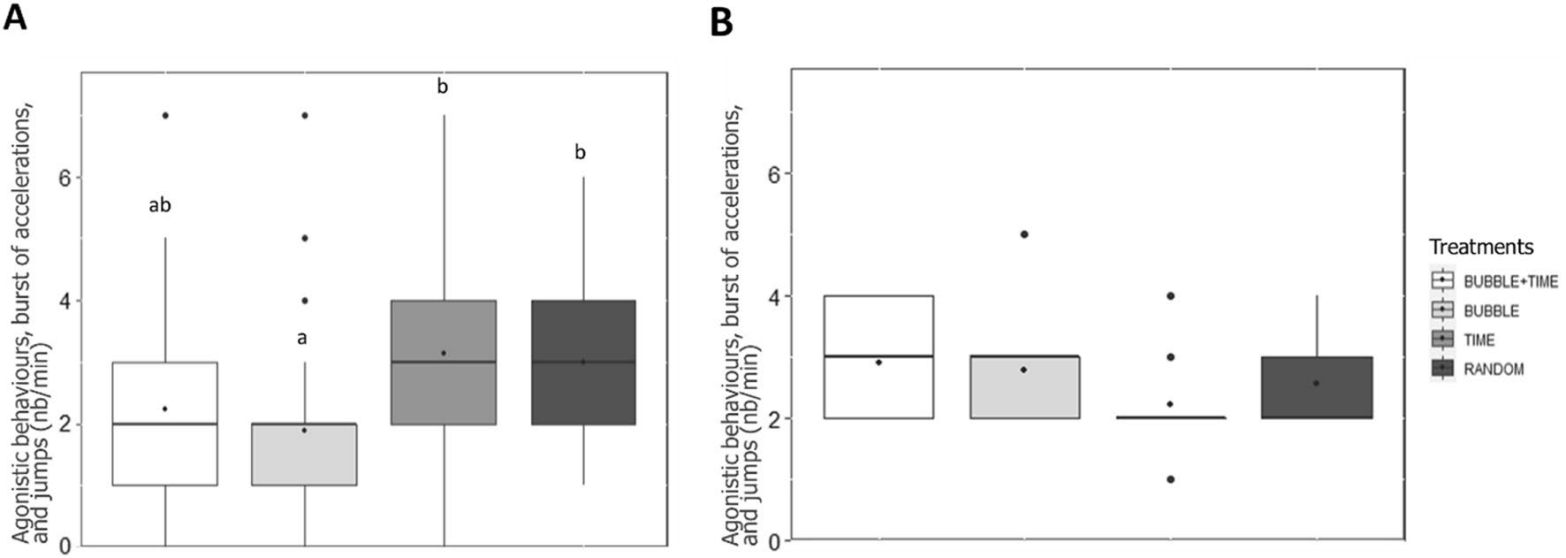
Median (quartiles: 25%, 75%) number of agonistic behaviours, burst of accelerations, and jumps scored per minute during (**A**) the 6-min period following each of the three feed omission occasions, when pooled, and (**B**) three neutral periods of 6 min free from feedings or bubbles on day 12. The black point inside boxes represents the mean value. Different letters indicate significant differences between treatments (*P* < 0.05; Tukey post-hoc tests).

Group activity analysed for the 6-min period that followed each omission was similar between treatments (LMM: *P* = 0.21). Overall group activity was significantly lower during omission 3 compared to omissions 1 and 2 (effect of the omission number: LMM: χ^2^ = 43.70, df = 2, *P* < 0.001; Tukey, *P* < 0.001). However, during this same period, agonistic behaviours, burst of accelerations, and jumps differed between treatments (GLMM: χ^2^ = 15.40, df = 3, *P* = 0.002; Fig. 6A). Fish from the treatment BUBBLE elicited significantly less agonistic behaviours, burst of accelerations, and jumps than fish from treatments TIME (Tukey, *P* = 0.003) and RANDOM (Tukey, *P* = 0.02). This treatment effect appeared to be specific of the days when omissions occurred. Indeed, on day 12, when observing agonistic behaviours, burst of accelerations, and jumps during neutral periods no difference was found between the four treatments (GLMM: *P* = 0.21; Fig. 6B).

### Individual fish’s emotional reactivity

All the measures scored during the novel-tank test (total distance moved, maximum swimming velocity, angular velocity, time spent in thigmotaxis, number of pellets eaten) were not different between groups, indicating thus a similar emotional reactivity among treatments (LMM: *P* > 0.05 and χ^2^ = 0.93, df = 3, *P* > 0.05 for all measured behavioural parameters).

## Discussion

The present work deepens our knowledge on how a farmed fish species, the rainbow trout, uses different environmental cues to predict feed delivery. We found evidence that rainbow trout anticipate feed delivery from as early as two weeks of conditioning and that their anticipation is expressed differently depending on the predictor type. Fish from the BUBBLE-TIME treatment were able to use both time and bubbles to predict the arrival of feedings as revealed by their higher pre-feeding activity before and after bubble diffusion during feed omissions. Fish for which feedings were signalled by bubble diffusion only (BUBBLE) were successfully conditioned to bubbles given their higher activity during feed omissions after bubble diffusion compared to fish that relied on temporal predictability only (TIME). In addition, BUBBLE fish expressed fewer agonistic interactions behaviours, burst of accelerations, and jumps following feed omissions than TIME and RANDOM fish. For fish that could only rely on temporal predictability for feedings (TIME), feed anticipation was manifested both in increased activity and in agonistic behaviours, burst of accelerations, and jumps before feedings. We found no effect of the treatments on the emotional reactivity of the fish or on their growth rate.

Over the twelve days of conditioning, the first results focussing on pre-feeding swimming activity did not allow us to observe that rainbow trout were successful in using bubbles as a predictor of feed delivery. We initially considered bubbles as a neutral signal to announce the feeding, but we observed that fish were strongly attracted to them regardless of the predictor, which may have masked any conditioning effects we might have observed over this period. Thus, we cannot rule out that bubble diffusion represents a salient positive stimulus that may have led to a high level of activity under all predictability conditions. This was probably due to a high arousal generated by bubbles, as indicated by the increased fish activity during bubbles compared with neutral periods for a signal we initially thought to be a neutral stimulus. Darwin^26^ argues that the strong link between stimuli and the emotion states they could elicit can either be inherited or learned by habit. From the first bubble diffusion, fish were immediately attracted to bubbles, and this remained true after 12 days of conditioning, potentially claiming for an inherent positive emotion-like state. Bubble diffusion would thus stand as a reward on itself and refer to the quadrant one (Q1) for positive high arousal affective states (e.g., excitement, joy) proposed for core affect by^27^ and reused by^28^ in the study investigating the emotional-like states induced by environmental stimuli in sea bream. Initial attraction to the signal announcing the event has also been reported in salmon and mammals conditioned to light before moving to the event area^25,29^. Lieberman^30^ states that this initial attraction to the conditioned stimulus follows the associative learning process which involves two phases: an initial substitution of the signal with feed (reflex response), and then a learning phase where the signal is associated with feed through anticipation and cognitive processes^15^. The first response is then supposed to be replaced by the second as part of the habituation process—a brain process that decreases the strength of a reflex upon multiple exposures to a stimulus^30^—but in our case, the trout continued to be highly active around the bubbles before feeding. Since rainbow trout were able to learn to predict feedings after a light signal in another study^31^, the behaviour of rainbow trout towards bubbles does not seem to be a reflex response, but rather a strong interest for bubbles. However, when considering activity in the bubble area after bubble diffusion in all treatments when feeding was omitted, we observed a higher level of activity in BUBBLE + TIME than in TIME and RANDOM, and BUBBLE fish also showed higher activity after bubbles than TIME fish. Since all the groups experienced bubbles during this analysis period, this result reveals that the higher activity is not simply due to agitation caused by bubble diffusion but that the BUBBLE + TIME and BUBBLE treatments have learned to use bubbles as a predictor of feedings after 14 days of conditioning (i.e., 70 trials). Our results are in accordance with studies on salmons which were successfully conditioned to receive feed after a light signal in 19 to 144 trials (19 trials:^11^, 72–144 trials:^13^, 56 trials:^22^). Bubbles conditioning was most effective when bubble diffusion was coupled with time. Indeed, activity before feedings was higher in BUBBLE + TIME than in RANDOM treatments by day 12 (after 60 trials), suggesting a more accurate conditioning when time is added.

Rainbow trout in the TIME and BUBBLE + TIME treatments showed greater pre-feeding activity (*i.e*., FAA, feed anticipatory activity) than fish in the BUBBLE treatment, suggesting that they were able to use time as a predictor. Rainbow trout from the BUBBLE + TIME treatment were, however, the most temporally conditioned to feeding, as evidenced by their higher FAA than in BUBBLE and RANDOM. To achieve this FAA, they required five daily feedings over 12 days, which is in agreement with previous studies showing that farmed fish require 15 to 60 days to elicit a stable FAA with one or two daily feedings (*Salmo salar*^12^; *Rhombosolea tapirina*^32^; *Dicentrarchus labrax*^10^). On day 12, FAA was evident by the 5th feeding (F3 on Fig. 3). The lack of difference between treatments prior to the first and third feedings certainly indicates that since all fish had an overnight fast, they were equally agitating because of hunger, and differences seemed more pronounced as the day progressed to become clearly noticeable at the last feeding of the day, when the fish had already been fed four times. The temporal-only predictability of feeding also resulted in more aggressive behaviours, burst of accelerations, and jumps before feedings compared to the other predictability conditions. Heydarnejad and Purser^33^ showed that agonistic behaviours can be a reliable indicator of feed anticipation in rainbow trout. Our results are in agreement with other salmonid studies that showed higher agonistic interactions when feeding was predictable over time than when it was unpredictable (*Salmo salar*^12^; *Oncorhynchus mykiss*^34^). TIME fish could only rely on their biological clock to predict feed delivery, and this time-window was likely less obvious to identify than a clear audible or visual signal, such as bubble diffusion^6^. In contrast, BUBBLE + TIME fish did not exhibit increased pre-feeding agonistic behaviours, burst of accelerations, and jumps, which even tended to be less frequent than in TIME, suggesting a specific role for bubble diffusion in establishing feed anticipation.

As mentioned before, feed omissions revealed that bubble diffusion as a predictor of feedings is effective in rainbow trout, despite its initial attractiveness, since BUBBLE + TIME and BUBBLE fish became more agitated in the bubble area after bubble diffusions than TIME and RANDOM fish. In addition to that, following feed omissions, levels of aggression, burst of accelerations, and jumps were lower for BUBBLE fish compared to TIME and RANDOM fish. This seems contradictory to previous studies showing that salmonids conditioned to associate a light with a reward exhibit strong aggressive behaviours when the reward is omitted^24,25^. These authors suggest that aggressive behaviours may be due to a state of frustration defined as ‘an aversive motivational state preceded by the omission of an expected reward’^35^. However, in these studies, aggressive behaviours were compared between dyads of conditioned fish that did not receive the expected reward and fish that did. Here, we compared aggression in conditioned and unconditioned fish. The lower level of aggressive behaviours, burst of accelerations, and jumps observed in BUBBLE fish after the omission of feed suggests these fish can adapt quick to, and are less stressed by environmental changes. It can also be assumed that bubbles alleviate fish frustration of not being fed due to their initial attractiveness coupled with their positive valence created by the positive conditioning and would divert the fish’s attention from the missed feeding, acting as a reward in itself. Conversely, RANDOM and TIME fish still displayed high levels of expectancy, expressed here as aggressive behaviours, burst of accelerations, and jumps, known to be indicators of poor welfare in farmed fish^36^. For TIME fish, this result is similar to what we observed before feedings. For RANDOM fish, this increase in aggressive behaviours after feed omissions compared to neutral periods could be explained by a constant state of expectation and frustration, since they had no cue to predict when their feedings would be available. These results are consistent with our hypothesis that occupational enrichment, by giving fish the ability to anticipate the events in their environment through a salient predictor, would lead to less aggression and thus a better welfare state, as has also been shown in mammals^6,8^.

In this experiment, we found similar growth rates between rainbow trout reared under the four conditions of feeding predictability. This is in agreement with results obtained in salmons for which temporal feed predictibility induced no effect on specific growth rate, compared to fish reared under unpredictable scheddules for the same experimental duration as ours (15 days)^12^. This may be explained by this short experimental period, as better growth rate was found after 30 days of temporally predictable feedings in sea bream^9^, 60 days in seabass^10^ and 20 weeks in Atlantic cod^37^. However, even for a 60-day experimental duration in salmon, light-signalled predictable feedings did not induce differences in growth, condition factor, or feed conversion ratio compared to salmons reared under unpredictable feeding conditions^16^. Here, giving rainbow trout the opportunity to predict feed delivery for 14 days, regardless of the type of predictor used, had no influence on growth parameters.

One of our initial assumptions was that the ability of fish to predict events occurring in their environment would improve their emotional reactivity and allow them to better cope with unexpected situations. However, our results showed that fish emotional reactivity, or their fear-responses when individually introduced into a novel environment, were similar between treatments. Emotional responses depend on how the animal perceives its environment (threatening, neutral, stimulating) and rearing conditions can impact these behavioural responses^38–40^. For example, rearing conditions where welfare was supposed to be good were linked to greater motivation to explore in fish tested for emotional reactivity^41^. Also, in a recent study, rainbow trout previously reared in a complex and stimulating habitat spent more time exploring the new environment when subjected to the novel-tank test and exhibited less erratic swimming compared to fish reared under standard conditions^42^. This suggests that good welfare conditions may lead to bold phenotypes and lower fearfulness measured in the novel-tank test. The lack of difference between our different predictors could be explained by high quality water (spring water, constant temperature) levelling out more subtle differences between treatments and/or by the presence of the bubbles which could have acted as an enrichment generating in itself a positive affective state for fish from each treatment, since they were all equally exposed to this stimulus. As reported by^27^ and^28^, the excitement caused by bubble diffusion could reflect positive affective states at high arousal levels. Environmental enrichment designed to increase habitat complexity was shown to generate positive effects on a variety of fish species^43^. A control group without bubble diffusion in our study would therefore have been beneficial in helping us clarify the role of bubbles as a potential enrichment rather than a neutral stimulus. To our knowledge, this is the first study highlighting that farmed fish are attracted to bubbles, other than for their oxygenating properties, for which bubbles are often released in fish aquaria. Bubbles provide visual, tactile, and acoustic stimuli that can be perceived simultaneously by all fish in the tank, which is why we chose bubble diffusion as the conditioned stimulus in the first place. Even if a sound as a CS succeeded in conditioning fish to feeding^20,21^, acoustic stimulation on its own seem to generate a flight response as shown in salmons when provided with an infrasound^44^ or in goldfish provided with a white noise^45^. Light conditioning for feeding was the most studied CS^13–17^ and could have represented a good option in our study as well. However, sharks (*Heterodontus portusjacksoni*) conditioned to receive feed after bubble diffusion showed higher anticipatory behaviours, manifested by movements and bites towards the CS, and higher retention memory than sharks receiving feed after a light signal for a same conditioning procedure^46^. The choice of the CS thus appears essential to consider for an effetive conditioning, privileging a salient stimulus.  Furthermore, by providing tactile stimulations, which have been shown to decrease stress levels in the surgeonfish *Ctenochaetus striatus*^47^, and/or by representing small objects to play with as has been observed in few fish species^48,49^, the sequenced diffusions of bubbles in the tank could perhaps represent a physical enrichment for rainbow trout, and their impact on fish welfare would deserve further investigation.

## Conclusions

We found that rainbow trout are able to anticipate feed delivery after 12 days of conditioning with time as the only predictor. TIME fish exhibited more frequent aggressive behaviours, burst of accelerations, and jumps when anticipating feedings, suggesting that using time alone as a feeding predictor in husbandry practices may be detrimental to fish welfare. The combination of temporal and signalled predictability elicited the highest conditioned response in fish and the level of pre-feeding aggression tended to be lower than in the TIME treatment. When feedings were signalled solely by bubble diffusion, fish were also able to associate bubbles with feed, as evidenced by their increased activity in the bubble area when feed was omitted after bubble diffusion compared to TIME. Also, in the BUBBLE treatment, fish exhibited fewer agonistic behaviours, burst of accelerations, and jumps before feedings and during feed omissions than in the temporal predictability condition. Our results suggest that bubbles may alleviate fishes’ frustration of not being fed by diverting their attention. Indeed, we found that bubbles were highly attractive regardless of the treatment considered. By acting as both an occupational (as a feeding predictor) and physical (as an attractive element) enrichment, predictability of feeding through bubble diffusion could represent an interesting approach to improve rainbow trout welfare in farms.

## Methods

### Ethics statement

All experimental procedures were carried out in strict accordance with the European Directive 2010/63/EU on the protection of animals used for scientific purposes. The study design was approved by the Ethics Committee in Animal Experiment of Finistère and have received authorization from the French Ministry of Education, Research and Innovation under the agreement number 2020-C2EA74-VC-01-V2. This study was carried out in compliance with the ARRIVE 2.0 guidelines.

### Animals and maintenance conditions

Animals were female triploid rainbow trout (*Oncorhynchus mykiss*) fertilized and reared at INRAE-PEIMA fish farming experimental facility (https://doi.org/10.15454/1.5572329612068406E12) from a delayed autumnal strain. At 100 days post fertilization (dpf), 1872 fish (2.71 g mean weight) were randomly chosen and weighed under anaesthesia (50 mg/L tricaine and 50 mg/L sodium bicarbonate) before being equally distributed into 12 rearing tanks (156 fish/tank) supplied with spring water. Tanks were uniform in colour (grey opaque walls), in size (70 × 70 cm), in volume of water (196 L) and in water flow (one renewal per hour). All tanks were equipped at both sides with a yellow light bulb (Leds 4000 K, 9.6 W, ELVADIS) controlled by a programmer panel to provide a 12L:12D photoperiod, a 35% light intensity, and a progressive lighting over 15 min. For bubble diffusion, each tank was provided with an air diffuser (4 × 6 mm PVC transparent air tubes attached to the end of the water inlet pipe) and two air pumps supplied six air diffusers at the same time throw a 6-valve low pressure air distributor. The valves of the dispensers were adjusted so that the bubble size was equal in each tank. Water temperature remained constant at 12 ± 0.2 °C and density was maintained below 20 kg/m^3^ throughout the experiment. Ammonia, nitrite and nitrate concentrations were checked at the beginning and at the end of the experiment and were always lower than 0.27, 0.00 and 35.90 mg L^-1^, respectively. From this stage (100 dpf), fish were fed five times a day, in equal amounts, with extruded and commercial flowing pellets (60% protein, 33% lipid, 7% starch, 1.1 mm pellets, BioMar, France) in accordance with fish growth rate. The feed was delivered by ARVOTEC feeders at precise schedules using the computer-controlled Imetronic software (version 2008). Rearing tanks were all equipped with a video-surveillance camera (Full HD: 1920 × 1080 px, 105°, VIZEO—Adrien Alarme) installed above each tank allowing continuous recordings.

### Conditions of fish raised with different possibility to predict feeding

**Figure 7:**
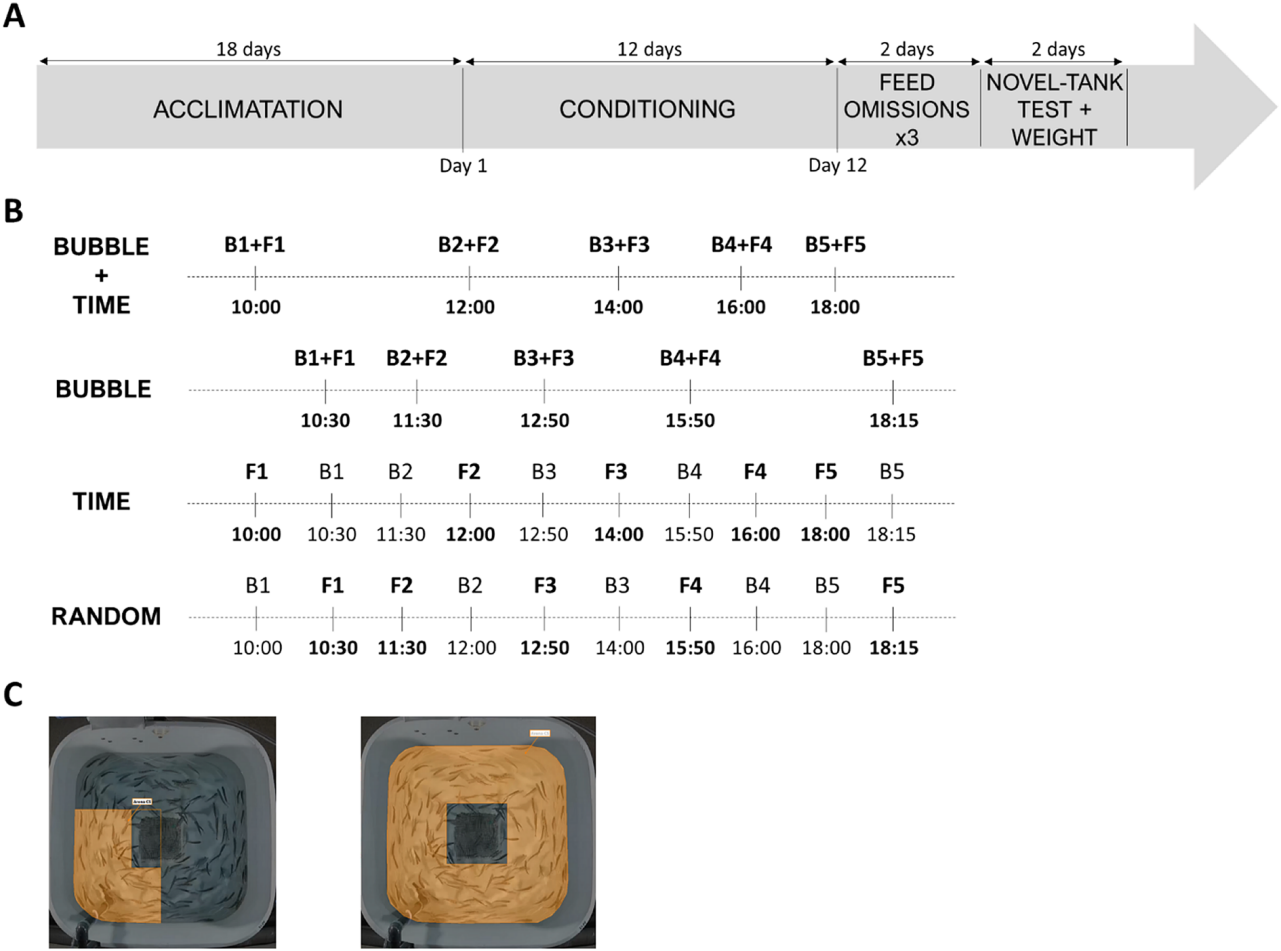
(**A**) General schedule of the experiment with a first period of acclimatation to the environment, the conditioning involving the four different predictability conditions of feeding between day 1 and day 12, then the three feed omissions performed in all treatments, and the novel tank-test after which growth parameters were recorded; (**B**) Example of a day during conditioning for each treatment, with BUBBLE + TIME based on temporal + signalled predictability of feeding (based on time and bubble diffusion), BUBBLE based on signalled predictability alone (based on bubble diffusion), TIME based on temporal predictability alone (based on time of day), and RANDOM corresponding to unpredictable feedings (random feeding delivery). B1 to B5 represent the five daily bubble diffusions, and F1 to F5 the five daily feedings; (**C**) Areas used for fish behaviour analyses (on the left: the bubble area where bubbles were diffused; on the right: the whole tank).

After an acclimation period of 18 days, four feeding predictability treatments were applied (three tanks per treatment containing 156 fish each) during twelve days (Fig. 7A). In each treatment, fish received each day five diffusions of bubbles (during 15 s each) and five feedings (total of 60 sequences of bubbles and feedings) (Fig. 7B).

1. Treatment BUBBLE: a signal of 15 s of bubble diffusion was systematically followed 5 s later by feeding (20-40 s). For this treatment, daily feedings were delivered each day between 9.45am and 7.20 pm according to a manually randomized schedule with a minimum of 15 min and a maximum of three hours between two feedings to avoid any starvation or digestion issues.
2. Treatment TIME: the occurrence of daily feedings could be predicted by the time of the day. Each day, fish received systematically their feed at the same time schedules: 10am, 12am, 2 pm, 4 pm and 6 pm. Fish from this condition also received bubbles five times per day, at the same time schedule as the one used for treatment BUBBLE but systematically separated from at least 15 min from feedings.
3. Treatment BUBBLE + TIME: both bubbles and time were predictive of feed delivery. Fish received daily feedings at the same time schedules as treatments TIME. Each feeding was also systematically preceded by 15 s of bubbles (as treatment BUBBLE).
4. Treatment RANDOM: neither bubbles nor time were predictive of feed delivery. Daily feedings were delivered according to the same randomized schedule as the one used for treatment BUBBLE. The bubbles were systematically delivered separated from at least 15 min from feedings.

### Group behaviours during the 12 days of conditioning

Previous studies showed that fish activity was a relevant behaviour as an indicator of anticipation of feed delivery^50^. Thus, group activity in the rearing tank was automatically analysed by EthovisionXT® software (v. 15.0.1418) (Noldus, Wageningen, The Netherlands) (Table 1). Activity was given as a proportion of pixels that changed intensity between two successive images (25 images per second). Only pixels within the defined area (= 100%) are taken into consideration. As a result, activity data represent global activity of the group of individuals. Data are given as percent of activity (%). Behavioural recordings were conducted at three different feedings or bubble sequences (1st, 3rd, and 5th sequences of the day) on the 1st and the 12th days of the conditioning to examine its progression over the experiment. Depending on the period targeted, analyses were performed into the bubble area or into the whole tank (Fig. 7C).

**Table 1:** Behavioural parameters used for observations during the 12 days of conditioning and feed omission tests.

Behaviour	Observation method	Definition
Activity	Instantaneous sampling (current activity at preselected moments in time) Ethovision XT software	Activity is calculated as a percent by taking every pixel in a given area and comparing it with the previous image. If all pixels remain the same, there is zero activity. If all pixels are different, there is 100% activity. If the animal is moving and increases its velocity (whilst keeping the same shape) there will be an increase in activity, because the pixels belonging to the animal are increasingly different as it moves faster
Agonistic behaviours	Continuous sampling (occurrence)	Bite attempts and chases through the tank. This parameter was counted when a fish moved away from its initial position with a propulsive tail stroke, immediately followed by another fish^52^. An occurrence was considered to end when the fish interrupted the behaviour for at least 5 s
Burst of accelerations	Continuous sampling (occurrence)	Sudden movement away from the initial position with propulsive tail strokes, without previous agonistic interaction^53^. An occurrence was considered to end when the fish interrupted the behaviour for at least 5 s
Jumps	Continuous sampling (occurrence)	Movement towards the water surface with sudden emergence of at least half of the body out of the water^53^

Fish ability to use bubble diffusion as a predictor of feed delivery was assessed by analysing their activity during the 15-s sequence of bubble diffusion added to the 5 s of time gap that followed. This analysis was performed in the bubble area. Fish activity was also analysed during neutral periods (i.e., 6-min period with no feed delivery or bubble diffusion) in the bubble area.

To assess whether fish could use time as a predictor of feed delivery, fish activity was analysed for the 6 min that preceded feedings (for treatments TIME and RANDOM) or bubble diffusions (for treatments BUBBLE and BUBBLE + TIME). Previous studies showed that agonistic behaviours, burst of accelerations, and jumps are observed when fish anticipate feedings relying on time as the main predictor^33^. In this aim, frequency of agonistic (bites, chases) behaviours, burst of accelerations (when fish crossed more than the half of the tank with high speed) and jumps were indiscriminately grouped and counted in the whole tank during the 6-min period (Table 1). An occurrence was considered to end when the fish interrupted the behaviour for at least 5 s.

### Feed omission tests

To assess whether fish could associate bubbles to feed delivery after 12 days of conditioning, three feed omissions were subsequently performed (no feed was delivered at the expected feeding moment): one on day 13 and two on day 14 (Fig. 7A). By separating omission trials with one conditioned trial, we maintained the associative value of the conditioned and unconditioned stimuli, similarly to what has been done in previous studies^24,25,51^.

During each omission, group activity (Table 1) was analysed during a period of time that included the 15 s of bubble diffusion, the 5 s of time gap before the expected feed and 25 s after the omission (totalling 45 s of observation). For treatments BUBBLE and BUBBLE + TIME, the last period of 25 s corresponds to the moment when feed was supposed to be delivered. For treatments TIME and RANDOM, the period of 25 s started 5 s after the end of bubbles. Similar to the conditioning phase, this analysis was performed in the bubble area.

As the omission of an expected reward is often characterized by an increased activity and aggressions in salmonids^24,25,51^, the total number of agonistic behaviours, burst of accelerations, and jumps (total/min) as well as fish activity (in %) in the whole tank were also scored during a 6-min period that followed each omission (Table 1). Total number of agonistic behaviours, burst of accelerations, and jumps (total/min) was also analysed during neutral periods (i.e., 6-min period with no feed delivery or bubble diffusion) in the whole tank on the day before the feed omissions (day 12) to detect any specificity due to feed omissions.

### Individual fish emotional reactivity

The emotional reactivity of an individual animal refers to its propensity to react to emotion-provoking stimuli such as suddenness and novelty^37^. Considering the emotional reactivity of an animal is therefore crucial for assessing how it perceives its environment (in a threatening way or not), its ability to cope with environmental changes, and thereby its welfare state. Fish emotional reactivity was evaluated after 12 days of conditioning on days 15 and 16 (Fig. 7A), in a novel-tank test following Colson et al.^53^. Ninety-six fish (24 fish/treatment and 8 fish/tank) were randomly netted and introduced individually into a novel tank (68 × 33 × 32 cm). Four fish were netted simultaneously from the same rearing tank and introduced into four separate test tanks at the same time. At the end of the test, another tank was sampled and so on until the 12th tank. Each rearing tank was sampled only once a day to avoid any difference in stress between the tested fish. The next day, four new fish were collected from the first rearing tank and then the remaining 11 tanks were collected in the same order as the previous day. Fish were all fed the day before the test with 3/5 of the feed ration in one feeding so that the tested fish could experience the same fasting interval. Bubble diffusions were stopped the day before testing. Water from the tested tanks was changed every fourth batch of fish tested. Behavioural responses were video recorded for 28 min. We analysed the first 20 min, by 5 min time-bins with the Ethovision ® XT software. At 23 min, individuals automatically received 80 feed pellets (approximately 100 mg) and the remaining pellets were counted after 5 min to measure a possible inhibition of feed intake (anorexia) following a stressful situation (novel tank exposure). Indeed, feed consumption recovery after an acute stressor is a classical stress indicator in fish^37^. The following behavioural parameters resulting from the automatic tracking of each individual were scored: total distance moved (in cm), maximum swimming velocity (in cm/s), angular velocity (in °/s) (i.e., erratic swim), and time spent (in %) in the border zone (i.e., thigmotaxis).

### Weight and size of fish

Fish body weight (W) and length (L) were measured after the novel-tank test under anaesthesia (50 mg/L tricaine and 50 mg/L sodium bicarbonate). For each fish, the condition-factor was calculated as followed: K-factor = 100 (W/L^3^).

### Statistical analysis

All tests and graphs were performed using the 1.4.1717 version of RStudio and were plotted using the packages ggplot2, ggthemes, and effects.

#### Group activity analysis

Group activity was analysed by performing linear mixed-effects models (LMER)s, with the lme4 package. Group activity (given in % by Ethovision) during the 6 min that preceded feed was analysed with treatments (BUBBLE + TIME, BUBBLE, TIME, RANDOM) and feedings number (feeding 1, 3 or 5), and their interaction, as fixed factors. Group activity during bubble diffusion (15 s bubbles + 5 s gap) was analysed with treatments and bubble diffusion number (diffusion 1, 3 or 5), and their interaction, as fixed factors. Rearing tanks were considered as random factors. The following LMER was used: lmer(Mean.activity ~ Treatment*Trial.number + (1|Rearing tank). During feed omissions tests, group activity was analysed with treatment and omission number (1, 2 or 3), and their interaction, as fixed factors. The following LMER was used: lmer(Mean.activity ~ Treatment*Omission.number + (1|Rearing tank). Similar to the previous model, rearing tanks were added as random factors.

The day (day 1 or day 12) was not included as a fixed factor into the models since data given by Ethovision did not account for fish growth between days 1 and 12 of the experiment, resulting in more pixel changes on day 12 compared to day 1.

All data were checked for normality before running a model and the dispersion of residuals was subsequently checked. If data did not meet the assumptions for parametric statistics, data transformation were applied else by inverse, log- or log10-transformation. For each model, the effects of fixed factors on each variable were evaluated using the analysis of deviance table with ANOVA of type III. Significant effects of fixed factors and their interaction are presented in the results section.

#### Agonistic behaviours, burst of accelerations, and jumps analysis

Agonistic behaviours, burst of accelerations, and jumps occurrences over the 12 days of conditioning were analysed by using generalized linear mixed-effects models (GLMER)s considering a Poisson family with log function. Group behaviours during the 6 min that preceded feed and the 6 min during neutral periods were analysed with treatments (BUBBLE + TIME, BUBBLE, TIME, RANDOM), days (day 1, day 12), and their interaction, as fixed factors. Rearing tanks were considered as random factors. The following GLMER was used: glmer(Mean.activity ~ Treatment*Day + (1|Rearing tank), family = poisson(link = “log”)).

For feed omissions tests, agonistic behaviours, burst of accelerations, and jumps occurrences were analysed for the 6 min following feed omissions and the 6 min during neutral periods with treatments (BUBBLE + TIME, BUBBLE, TIME, RANDOM), omission number (1, 2 or 3) or period number for neutral periods (1, 2 or 3), and their interaction, as fixed factors. Rearing tanks were considered as random factors.by using this GLMER: glmer(Mean.activity ~ Group*Omission.number + (1|Rearing tank), family = poisson(link = “log”)).

For each model, the effects of fixed factors on each variable were evaluated using the analysis of deviance table with ANOVA of type III. Significant effects of fixed factors and their interaction are presented in the results section. When significant, only the effect of the interaction of fixed factors is presented in the results section.

#### Emotional reactivity

The analysis of the emotional reactivity consisted in testing the effect of the treatment on each variable (total distance moved, swimming velocity, angular velocity, time spent in thigmotaxis). LMERs or GLMERs considering a gamma family in case of non-normally distributed data, were used for all variables. The rearing tank was defined as a random factor. We used a chi-square test to analyse the effect of the treatment on the number of pellets eaten at the end of the novel-tank test.

Fish weight, length, and K-factor were compared between treatments using One-way ANOVA.

When significant effects were found, post-hoc analyses were performed using HSD-Tukey tests. P-values < 0.05 were considered statistically significant for all statistical analyses.

## Supplementary Information

Supplementary Information.
